# B cells in tumor draining lymph nodes act as efficient antigen presenting cells in cancer patients

**DOI:** 10.1186/2051-1426-3-S2-P65

**Published:** 2015-11-04

**Authors:** A Ali Zirakzadeh, David Krantz, Malin Winerdal, Christian Lundgren, Ciputra Adijaya Hartana, Emma Ahlén Bergman, Johan Hansson, Benny Holmström, Alexander Sidiki, Janos Vasko, Markus Johansson, Per Marits, Amir Sherif, Ola Winqvist

**Affiliations:** 1 Karolinska Institutet, Stockholm, Sweden; 2 Centre for Research and Development, Faculty of Medicine, Uppsala University, Uppsala, Sweden; 3 Department of Urology, Uppsala University Hospital, Uppsala, Sweden; 4 Department of Urology, Länssjukhuset Ryhov, Jönköping, Jönköping, Sweden; 5 Department of Medical Biosciences, Pathology Umeå University, Umeå, Sweden; 6 Departemnt of Urology, Sundsvall Hospital, Sundsvall, Sweden; 7 Department of Surgical and Perioperative Sciences, Urology and Andrology, Umeå University, Umeå, Sweden

## Introduction

Overall Survival of patients with muscle invasive urothelial bladder cancer MIBC remains around 50% (5 years), albeit some improvements by combining neoadjuvant chemotherapy with radical surgery. Our previous work has demonstrated that *in vitro* expansions of sentinel node-acquired autologous tumor specific CD4^+^ T cells are promising for adoptive immunotherapy [1]. In order for naive T helper cells to become activated, they need effective APCs, presenting tumor antigens. In another study, we observed that B cells in cancer patients were tumor antigen experienced and from their phenotypes we suggested a CD4^+^ T cell dependent anti-tumoral response [2]. In this study, we report a flow cytometric investigation of tumor draining lymph node (sentinel node) derived B cell activation by autologous tumor extract in patients with MIBC.

## Methods

Sentinel nodes (SNs) from 28 patients with MIBC were detected by a Geiger meter at cystectomy after peritumoral injection with radioactive isotope. Lymphocytes were isolated from freshly received SNs where they were stimulated with autologous tumor extract in a sterile environment. After cultivation for 7 days, the cells were analyzed by multi-color flow cytometry using FASCIA (Flow cytometric Assay of Specific Cell-mediated Immune response in Activated whole blood).

## Results

Patients displayed an increased B cell activation in SNs after stimulation with autologous tumor extract compared to when SN acquired lymphocytes were stimulated with autologous extract of macroscopically non-malignant bladder. CD4^+^ T cells from SNs were activated and formed blasts after co-culture with SN acquired B cells in the presence of tumor antigen. However, CD4^+^ T cells were not activated and did not blast when co-cultured with B cells incubated with HLA-DR-blocking antibodies. This indicates antigen presenting ability of SN acquired B cells.

## Conclusions

We demonstrate sentinel node acquired B lymphocytes can be activated in culture upon stimulation with autologous tumor extract but not with extract of non-malignant epithelium of the bladder, after 7 days. Lower number of sentinel node acquired CD4^+^ T cells cultured with HLA-DR blocked CD19^+^ cells in presence of tumor antigen, indicate functional antigen presenting ability of B cells in sentinel nodes. The role of B cells as APCs in human T cell anti-tumoral response should be further explored, as well as their usefulness in adoptive immunotherapy.
